# Cotinine Plus Krill Oil Decreased Depressive Behavior, and Increased Astrocytes Survival in the Hippocampus of Mice Subjected to Restraint Stress

**DOI:** 10.3389/fnins.2018.00952

**Published:** 2018-12-17

**Authors:** Cristhian Mendoza, Nelson Perez-Urrutia, Nathalie Alvarez-Ricartes, George E. Barreto, Raquel Pérez-Ordás, Alex Iarkov, Valentina Echeverria

**Affiliations:** ^1^Universidad San Sebastián Fac. Cs de la Salud, Concepción, Chile; ^2^Departamento de Nutrición y Bioquímica, Facultad de Ciencias Pontificia Universidad Javeriana, Bogotá, Colombia; ^3^Instituto de Ciencias Biomédicas Universidad Autónoma de Chile, Santiago, Chile; ^4^Facultad de Ciencias de la Actividad física y el deporte Universidad Pablo de Olavide, Sevilla, Spain; ^5^Research & Development Service, Bay Pines VA Healthcare System Bay Pines, FL, United States

**Keywords:** depression, cotinine, anxiety, restraint stress, krill oil, astrocytes

## Abstract

Restraint stress (RS) is a condition affecting millions of people worldwide. The investigation of new therapies to alleviate the consequences of prolonged RS is much needed. Cotinine, a nicotine-derivative, has shown to prevent the decrease in cerebral synaptic density, working memory deficits, anxiety, and depressive-like behavior after prolonged restraint stress (RS) in mice. Furthermore, post-treatment with cotinine reduced the adverse effects of chronic RS on astrocyte survival and architecture. On the other hand, the nutritional supplement krill oil (KO), has shown to be beneficial in decreasing depressive-like behavior and oxidative stress. In this study, in the search for effective preventative treatments to be used in people subjected to reduced mobility, the effect of co-treatment with cotinine plus KO in mice subjected to prolonged RS was investigated. The results show that cotinine plus KO prevented the loss of astrocytes, the appearance of depressive-like behavior and cognitive impairment induced by RS. The use of the combination of cotinine plus KO was more effective than cotinine alone in preventing the depressive-like behavior in the restrained mice. The potential use of this combination to alleviate the psychological effects of reduced mobility is discussed.

## Introduction

Stress is generated when an individual is unable to cope with overwhelming physical or psychological demands. Although, the hormonal and behavioral changes that occur in response to threatening stimuli is crucial for survival and can be beneficial in recruiting adaptive responses to cope with a stressful situation, however, prolonged stress can result in maladaptive changes that may lead to mental illness, cognitive and motor deficits (Yehuda et al., 1998; Hammen, 2003; Luine et al., 2007).

In the clinical realm, the emotional and physical alterations induced by RS further diminish the quality of life of people with restricted mobility as stress/induced Neuroinflammation induce

depression (Iwata et al., 2013; Muscatelli et al., 2017). A meta-analysis including 354 studies, 18,374 individuals revealed that more than 70% of depressed individuals showed signs of stress such as elevated cortisol levels in plasma(Stetler and Miller, 2011).

Stress response when engaged for extended durations or activated by a traumatic event; it is linked to the dysregulation of the Hypothalamus-pituitary-adrenal (HPA) axis (Bauer et al., 2001). This dysregulation results in altered levels of glucocorticoid hormones and neurotransmitters in the brain and may lead to psychological depression (Hayase, 2011). For example, since the HPA axis has bidirectional relationships with the serotoninergic system its dysregulation can result in decreased levels of serotonin leading to depression and irritability. Also, neurons from the amygdala (AMY), which are responsive to the corticotropin-releasing hormone (CRH), project to the raphe nuclei, the main serotonin source to the forebrain. In addition, dysregulation of the HPA axis correlates with morphological alterations of the brain such as the reduction of hippocampal volume observed in individuals with major depressive disorder (MDD) and posttraumatic stress disorder (PTSD) (Sheline, 2000; Bonne et al., 2001; Czeh et al., 2001; Schmitz et al., 2002; Villarreal et al., 2002; Drevets et al., 2008; Felmingham et al., 2009; Apfel et al., 2011; Filipovic et al., 2011; Gonul et al., 2011; Teicher et al., 2012; Admon et al., 2013; Ahmed-Leitao et al., 2016).

Chronic stress has many other harmful effects on the brain including neuroinflammation, oxidative stress, microgliosis, reduced neurogenesis, and diminished numbers and architectural complexity of neurons. Chronic stress also evokes synaptic alterations including spine number and shape (Kassem et al., 2013; Bennett and Lagopoulos, 2014; Scharfman and MacLusky, 2014). In rodents, restraint stress (RS) affects spatial memory (Bowman et al., 2002; Kleen et al., 2006) and long-term potentiation, a cellular model of learning and memory processes, in the hippocampus (Pavlides et al., 2002). Such effects have been associated with the retraction of apical dendrites, as well as the loss of synapses in the CA1, CA3, and dentate gyrus (DG) sub-regions of the hippocampus (McEwen et al., 1997; Magarinos et al., 2011). These cellular changes and brain connectivity, contribute to the development of PTSD in people subjected to trauma and suffering with reduced mobility. As a result, several neurological symptoms appear such as working memory loss, impulsivity, aggressive behavior, and depression in traumatized individuals who developed PTSD (Tafet and Bernardini, 2003; Reagan et al., 2008; Conrad and Bimonte-Nelson, 2010; Luine, 2016; Moreira et al., 2016).

Numerous studies have emphasized the role of astrocytes in mediating brain homeostasis by supporting the blood-brain barrier function, sustaining neuronal energy metabolism (Stobart and Anderson, 2013), and neurotransmission (Schousboe et al., 1992). Furthermore, astrocytes modulate synaptic plasticity processes including synaptogenesis, neurogenesis, and learning and memory (Honsek et al., 2012; Bernardinelli et al., 2014; Haydon and Nedergaard, 2014). For these reasons, astrocytes are considered useful therapeutic targets for several neurological disorders (Garzon et al., 2016; Gonzalez-Giraldo et al., 2017).

Astrocytes have been classified according to their cellular morphologies (cell body size, and number, length, thickness, direction, and length of processes) and location, in two main subtypes, protoplasmic or fibrous (Sofroniew and Vinters, 2010). Protoplasmic astrocytes are found in the gray matter and present numerous stem branches that originate from several branching processes in a regular sphere-like distribution. Fibrous astrocytes have elongated processes and are present in the white matter (Sofroniew and Vinters, 2010). It has been proposed that the morphology of astrocytes can be a good indicator of their functions (de Filippis, 2011). Recently, Choi et al. studied the molecular and morphological changes of astrocytes induced by fear conditioning; the results showed a significant decrease of the immunoreactivity (IR) for the glial fibrillary acidic protein (GFAP) in the hippocampus of fear conditioned rodents (Saur et al., 2016). Our team found a similar reduction in the number of GFAP+ astrocytes in the CA1, CA3, and DG of the hippocampus after prolonged restraint stress (RS) in mice (Perez-Urrutia et al., 2017). Previous studies indicated that in the hippocampal formation posttreatment with cotinine normalized the number of GFAP+ astrocytes astrocyte and GFAP IR, in all hippocampal regions being the effect especially significant in the DG.

In this study, our goal was to assess the effect of co-treatment with KO plus cotinine on GFAP+ IR in the DG, and depressive-like behavior and working memory in a mouse model of prolonged RS. The results highlight the advantage of using cotinine plus a natural antioxidant nutrient to diminish stress-derived depressive behavior, cognitive impairment, and astrocytes abnormalities in the hippocampus after prolonged RS.

## Materials and Methods

### Animals

Two-month-old male C57BL/6J mice (obtained from the University of Chile), weighing 25-30 g, were maintained on a 12-h (h) light/dark cycle (light on at 07:00 h) with *ad libitum* access to food and water and maintained at a controlled temperature (25 ± 1°C). Upon arrival, mice were group housed and acclimated for 7 days before behavioral testing. Experiments were completed during the light period of the circadian cycle. Animal handling and care were performed in compliance with the Guide for the care and use of laboratory animals adopted by the National Institute of Health (USA), according to a protocol approved by the ethical committee of the Universidad San Sebastian, Chile.

### Drug Preparation

Cotinine ((5*S*)-1-methyl-5-(3-pyridyl) pyrrolidine-2-one) (Sigma-Aldrich Corporation, St. Louis, MO, USA) was prepared by dissolving the powdered compound in sterile phosphate buffered saline (PBS). KO capsules (300 mg) were purchased from Walgreens (Superba, USA). Soft gels contain 300 mg KO (90 mg omega-3 fatty acids, 50 mg eicosapentaenoic acid, 24 mg docosahexaenoic acid, 130 mg phospholipids). Manufacturers provided no information about the Astaxanthin content in the soft gels.

### Experimental Groups and Drug Treatments

**Figure 1:**
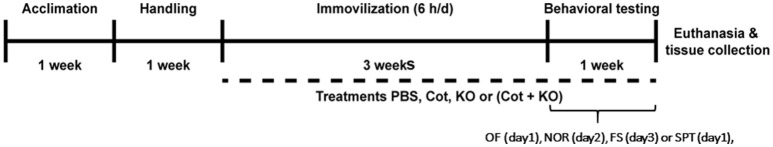
Experimental design. Mice were subjected to restraint stress 6 h/day for 21 days and co-treated with PBS, krill oil (KO), Cotinine (Cot) or Cot plus KO. After restraint and under continuing treatments, mice were tested for locomotor function, recognition memory using the novel object recognition test (NOR) and depressive-like behavior using the forced swim (FS) test and the sugar preference test (SPT).

Initially, the mice were acclimated for 1 week and subjected to handling to reduce stress. Then, mice were randomly divided into five groups (*n* = 8 mice/condition) and treated via gavage with 0.05 ml of vehicle (PBS, pH 7.4), Cotinine (Cot), KO or Cot plus KO dissolved in 0.05 ml of vehicle as detailed: (1) control non-restrained mice treated with vehicle; (2) restrained mice treated with vehicle; (3) restrained cotinine-treated mice (5 mg/kg in vehicle); (4) restrained KO-treated mice (143 mg/kg in vehicle); (5) restrained mice treated with cotinine (5 mg/kg) plus KO (143 mg/kg, in vehicle). Mice were treated via at the same time of the day, 30 min (min) before restraint and continuously until euthanasia. After 21 days (d) into treatments, mice were tested for behavior in the order from less stressful test to more stressful as indicated in Figure 1, with 24 of resting between tests to permit adequate resting of mice and to prevent interference between test. After the end of experiments, mice were euthanized using cervical dislocation by well-trained personnel.

### Behavioral Procedures

#### Restraint Stress

RS is a reliable method to model chronic stress that mimics the effects of restraint stress on brain functions and behavior in rodents (Pare and Glavin, 1986; Jaggi et al., 2011). Mice were gently introduced into 50-ml conical transparent plastic tubes (Corning Inc.) containing non-protruding perforations in both ends and in the walls to permit ventilation. Mice were kept inside these tubes, allowing slight movements, for 6 h a day for 21 d at room temperature (RT). After the daily restraint time, mice were returned to their home cages and permitted to move freely for the rest of the day. Following the 3 weeks of RS, mice were tested for locomotor activity and depressive-like behavior.

#### Open Field Test (OF)

**Figure 2:**
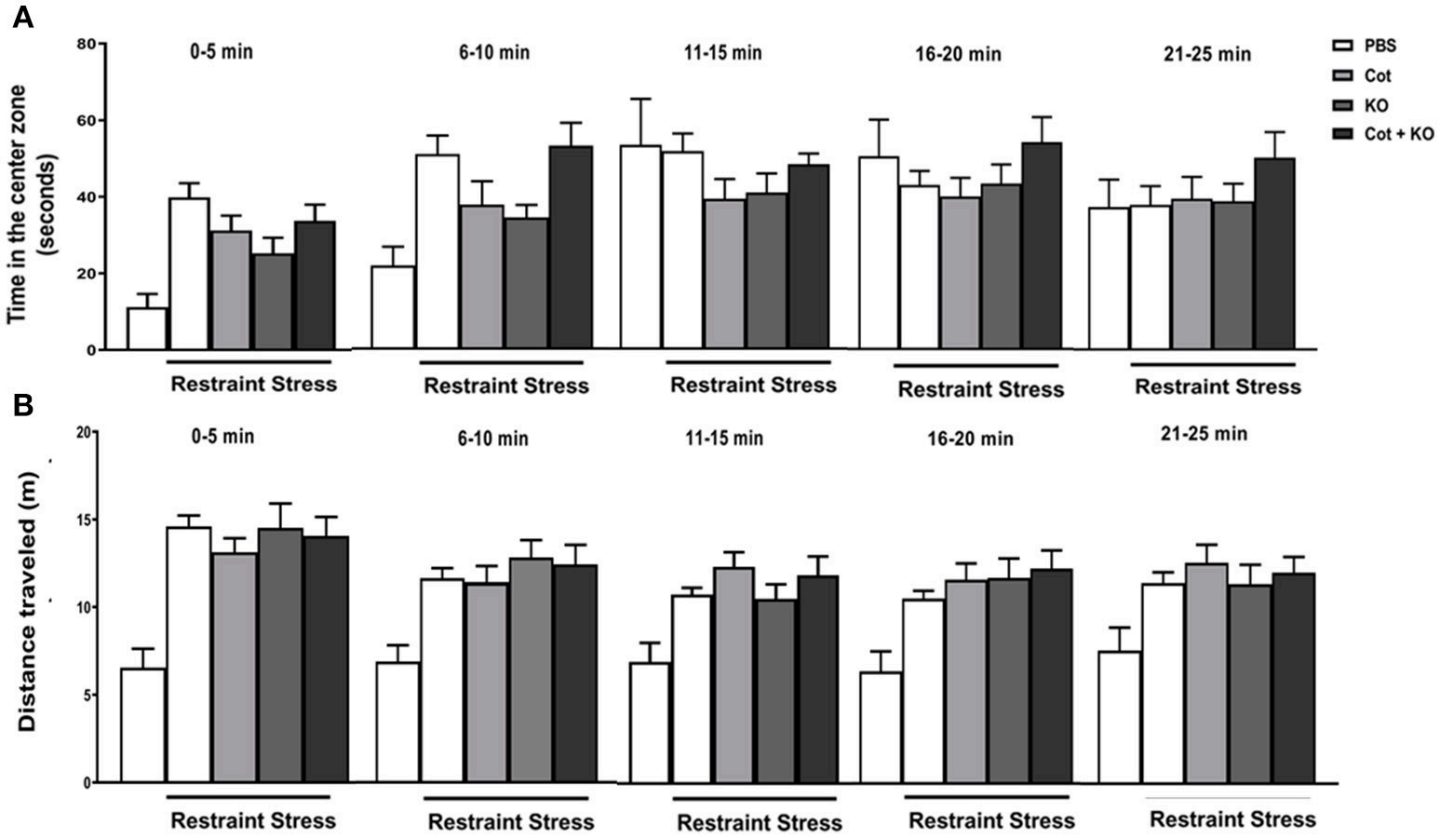
Co-treatment with cotinine and krill oil does not affect locomotor activity in mice. After prolonged restraint stress (RS) and co-treatment with vehicle (PBS), cotinine (Cot, 5 mg/kg), krill oil (KO, 143 mg/kg) or (Cot plus KO), mice were tested for locomotor activity in the open field test for 25 min. The results show the effect of RS and treatments on. Time in the center zone **(A)** and total distance travelled **(B)**.

The open field (OF) test (Belzung and Griebel, 2001) was conducted as previously described with minor modifications (Norcross et al., 2008) to identify changes in locomotor activity in response to stress and drug treatments. Mice were individually placed in a corner and permitted to freely explore an open square arena (40 × 40 × 35 cm) for 10 min (Figure 2A). Total distance traveled, speed and time spent in the center zone were measured under moderate lighting using the video tracking software (ANY-Maze, Stoelting Co.).

#### Forced Swim Test

**Figure 3:**
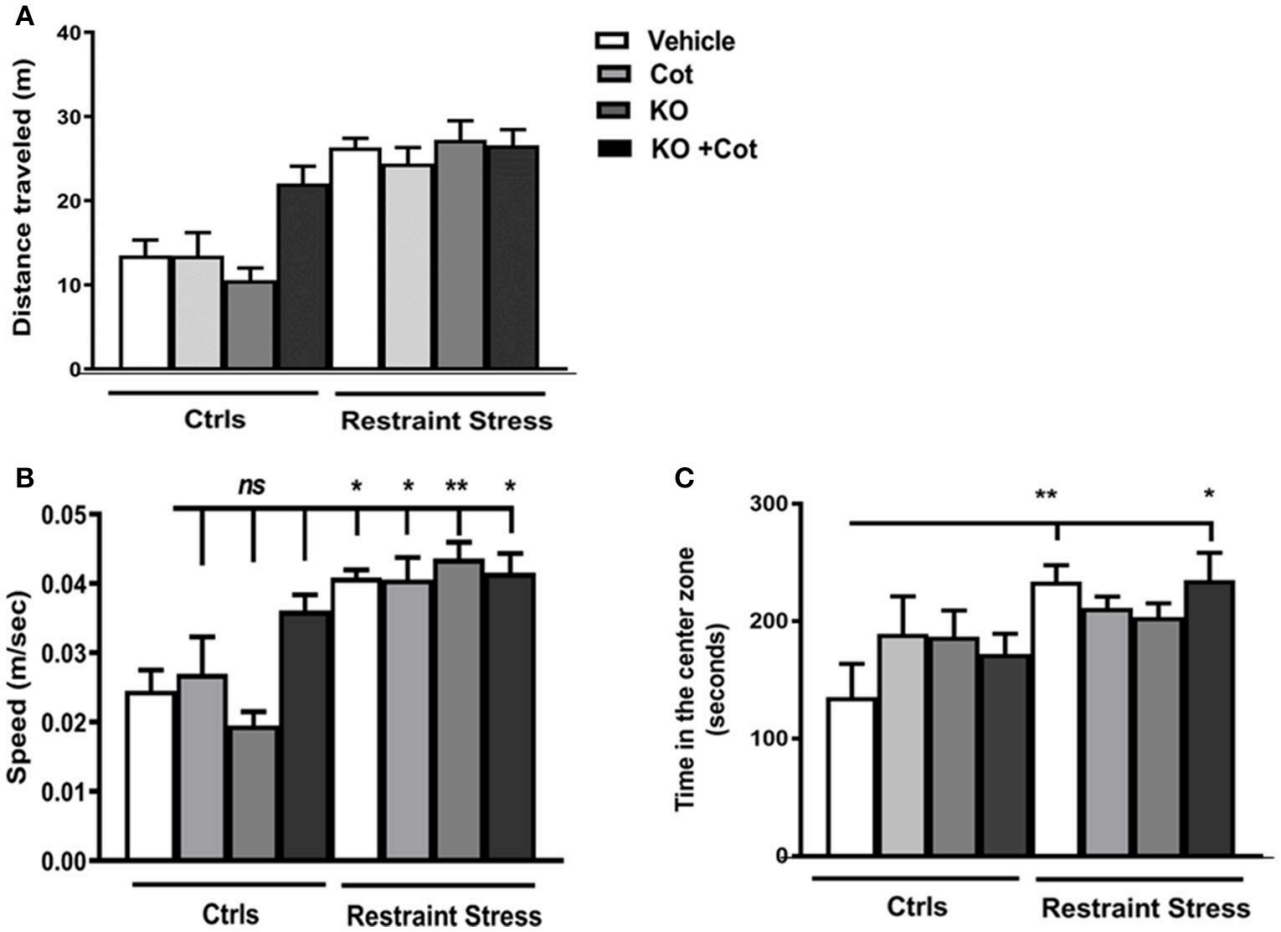
Co-treatment with cotinine and krill oil does not affect locomotor activity in mice. After prolonged restraint stress (RS) for 10 min and co-treatment with vehicle (PBS), cotinine (Cot, 5 mg/kg), krill oil (KO, 143 mg/kg) or (Cot plus KO). The results show that RS did not significantly affect locomotor activity in the stressed mice. **(A)** Total distance traveled. **(B)** Mean speed (meters/seconds) **(C)** Time in the center zone. *P < 0.05, **P < 0.01.

The forced swim (FS) is a broadly used task to assess depressive-like behavior in rodents (Dalla et al., 2010). The FS is performed introducing each mouse in the surface of a transparent and inescapable cylinder two-thirds filled with water at 26 ± 1°C (Figure 3A). Mice engage in periods of intense movement followed for increasing periods of immobility. The immobility time is considered an expression of depressive-like behavior. Immobility time is defined as no longer exhibiting any escape behavior, motionless or moving only to keep floating. Immobility time during a 5-min trial was recorded and quantified by two investigators blind to the treatment groups.

#### Novel Object Recognition (NOR)

**Figure 4:**
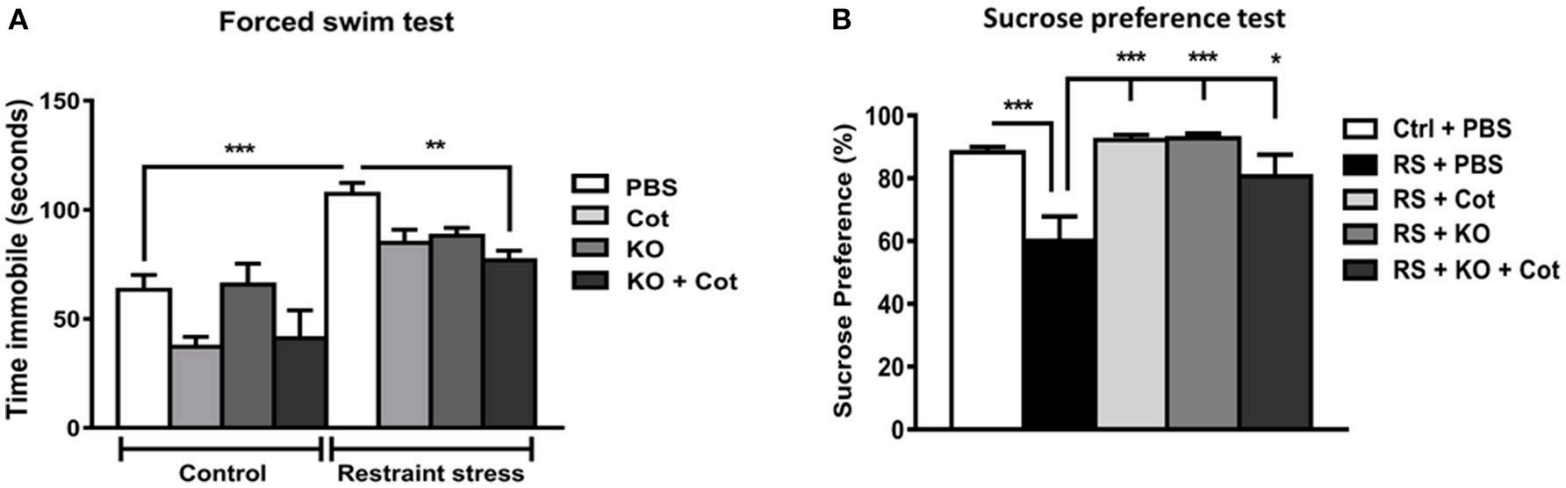
Co-treatment with cotinine plus krill oil prevented the restraint stress-induced depressive-like behavior in mice. After three-week restraint and co-treatment with vehicle (PBS), cotinine (Cot, 5 mg/kg) or krill oil (KO, 143 mg/kg), mice were tested for depressive-like behavior in the forced swim tests (5 min). **(A)** or the sucrose preference tests **(B)**. *P < 0.05, **P < 0.01, ***P < 0.001.

This task evaluates recognition memory, and it is based on the natural preference of rodents for novel objects when exposed to new and previously encountered objects (de Bruin and Pouzet, 2006). During the task, favored exploration of the novel object provides a measure of recognition memory. After a habituation step in a square arena (40 × 40 × 35 cm), each mouse was placed in the same arena but containing two identical transparent objects located equidistant to each other (familiarization phase) and led to explore the objects for 5 min (Figure 4A). Then, mice were returned to their cages and permitted to rest for 30 min. After resting, mice were placed back in the arena containing one of the familiar objects and a new object (Figure 4B). The time exploring the two objects was recorded for 5 min. Exploratory behavior was recorded and the time of exploration of each object was normalized for animal activity by calculating the exploration index (EI) that corresponds to the time spent by the mouse exploring one of the equal objects or the new object/ total time spent exploring both objects × 100%. The behavioral recording and analysis were performed using the (ANY-Maze, Stoelting Co.).

#### Sucrose Preference Test

The sucrose preference test was used to assess anhedonia, an indicator of depressive-like behavior. The sucrose preference test was performed as previously described (Serchov et al., 2016) with some modifications. The mice were habituated given two identical bottles with tap water in their cage. Then, mice were given free access to both, one with 2% sucrose solution and another with tap water for 3 days. The position of bottles was switched daily to avoid place preference in drinking behavior. Bottle weights were taken every day to determine solution intake. The preference for sucrose was calculated as a percentage of the volume of sucrose intake over the total volume of fluid intake and averaged over the 3 days of the test.

### Morphological Analyses of Astrocytes in the Dentate Gyrus

#### Brain Tissue Preparation

For the protein analyses, mice were euthanized, and brains removed. The left hemisphere of brains was dissected out to collect the regions of interest and quickly frozen for later biochemical analyses. For the immunohistochemistry (IHC) and fluorescent IHC (F-IHC) analysis, the right hemisphere of each mouse brain was placed in 4% paraformaldehyde in PBS pH 7.4 at 4°C for 24 h. The tissues were embedded in 2% agarose molds for vibratome sectioning. The region of interest was located using the Paxinos Atlas as a reference (Franklin and Paxinos, 1997), and serial sections of 20 μm (*n* ≥ 2/mouse) were collected using the Vibratome Leica VT1000S and placed on positively charged slides (Biocare Medical, Concord, CA).

#### Immunofluorescence and Confocal Microscopy

For the F-IHC, samples were washed three times for 7 min with Tris-buffered saline (TBS), pH 7.8. The primary antibody anti-GFAP (1:50, BioSB) was diluted in diluent buffer, containing TBS supplemented with 1% bovine serum albumin (BSA) and 0.2% Triton X-100 and incubated with the tissue sections overnight (ON) at 4°C. After three washes with TBS for 10 min, sections were incubated with the secondary antibody, Cy2-conjugated rabbit anti-mouse IgG (1:200, Jackson Immuno Research, Pennsylvania, USA) diluted in TBS containing 1% BSA for 2 h at RT. The samples were counterstained with Hoechst (1:1,000) and mounted with fluorescence mounting medium (Prolong, Invitrogen). Confocal z-stacks were acquired using an LSM 780 confocal microscope (Zeiss, Oberkochen, Germany), z-stacks were normalized to maintain a consistent signal intensity through the depth of the sample, confocal z-stack image series were superposed in maximum intensity projections by ImageJ (National Institute of Health, Bethesda, MA, USA) for the measurements.

#### Morphometric Analysis and Cell Counting

In each image, regions of interest (ROI) were defined on the dentate gyrus using free-hand drawing. For each ROI, the mean gray values (MGV), representing the area immunoreactive for GFAP, were measured. To measure GFAP immunostaining in the DG, maximum fluorescence intensity projections of confocal z-stacks acquired from sagittal brain sections were converted into 8-bit greyscale images with 256 scales (pixel intensity 0 corresponding to no signal and 255 to maximal signal) by Image J software. To calculate the area fraction of GFAP+ IR, binary images the immunoreactive area of the thresholded images was divided by the total of the ROI. For the GFAP+ cell counting, cell to be counted must have at least half of the cell nucleus visible on the edge of the ROI and cells to be included in the analysis must be not adherent to blood vessels.

#### Statistical Analysis

To analyze group and treatment effects differences in the means between groups one-way analysis of variance (ANOVA), and *post hoc* Dunnet's test was performed using the GraphPad Prism software to assess difference significance between groups. Differences were considered significant with *P* < 0.05.

## Results

### Effect of Krill oil and Cotinine on Locomotor Activity

To determine the changes in locomotor activity induced by co-treatments during RS, mice were tested using the OF test for 25 min. The results show that stressed mice showed higher time spent in the center zone than non-stressed controls in the first 10 min of the tests, but the differences diminished after this time between groups. The time spent in the center zone by the different groups was significantly different in the first 5 min of the OF test [*F*_(4, 63)_ = 6.42, *P* < 0.0003; Figure 2A]. At the contrary, the distance traveled show marked differences between non-stressed mice and all groups of stressed mice, independent of treatments (Figure 2B). Considering these results we proceed to analyze the changes in locomotor activity in the OF during the first 10 min of testing. One-way ANOVA analysis revealed significant differences in the distance traveled in the first 10 min [*F*_(7, 67)_ = 16, *P* < 0.0001; Figure 3A] and speed [*F*_(7, 76)_ = 8.91, *P* < 0.0001; Figure 3B]. There were no statistically significant differences in distance traveled or speed between non-stressed mice treated with vehicle, cotinine or KO. However, they showed significantly lower distances than non-stressed mice treated with KO plus cot (*P* < 0.05), and restrained mice treated with vehicle, cotinine, KO or KO plus Cotinine (*P* < 0.0001).

The increase in distance traveled was accompanied by a significant increase in speed in the mice subjected to RS when compared to non-stressed vehicle-treated mice (& RS, *P* < 0.05; & RS plus Cot *P* < 0.05; & RS, KO, *P* < 0.01; & RS, KO plus Cot, *P* < 0.05; Figure 3B). Also, the time spent in the center zone during the first 10 min of testing was analyzed, when anxiety behavior was more evident. The results showed that in the first 10 min there was a non-significant increase in time spent in the center zone between control non-stressed mice and the mice subjected to RS [*F*_(7, 67)_ = 2.11, *P* < 0.053] (Figure 3C). However, these differences were significant between treatment groups after 5 min of testing [*F*_(4, 67)_ = 9.17, *P* < 0.0001; (Figure 2A) with significant differences between non-stressed mice and stressed-mice independent of treatments (& RS, *P* < 0.0001; & RS plus Cot *P* < 0.001; & RS, KO, *P* < 0.001; & RS, KO plus Cot, *P* < 0.0001).

### Effect of Krill Oil and Cotinine on Depressive-like Behavior

To investigate the potential anti-depressant effects of cotinine, KO and KO plus cotinine when administered during stress, at the last day of RS mice were tested for depressive-like behavior using the forced swim test and sucrose preference test (Figure 4). A two-way ANOVA showed a significant impact of prolonged RS on the level of depressive-like behavior, expressed as a general increase in the immobility time during the FS test by the restrained mice when compared to control mice [*F*_(1, 38)_ = 15.35, *P* = 0.0004]. Also, this analysis revealed a significant effect of treatments on depressive-like behavior [*F*_(3, 38)_ = 5.23, *P* = 0.004]. Multiple comparison tests showed that mice subjected to RS and co-treated with Cot (*P* < 0.05), KO (*P* < 0.05) or KO plus cotinine (*P* < 0.01) showed lower levels of immobility than vehicle-treated restrained mice (Figure 4A).

To corroborate the antidepressant effect of treatments, a sucrose preference tests were performed. Also, the sucrose preference test revealed significant differences between treatments. One-way ANOVA revealed a significant effect of chronic stress in sucrose preference [*F*_(4, 28)_ = 8.65, *P* = 0.0001]. Multiple comparison tests showed significant decrease in sucrose preference in the vehicle-treated restrained mice when compared to vehicle-treated non-stressed mice. However, treatments showed antidepressant effects and the stressed mice treated with cotinine (*P* < 0.001), KO (*P* < 0.001) or cotinine plus KO (*P* < 0.05) showed significantly lower levels of anhedonia and higher sucrose consumption than the restrained mice treated with vehicle (Figure 4B).

### Effect of Krill Oil and Cotinine on Recognition Memory

**Figure 5:**
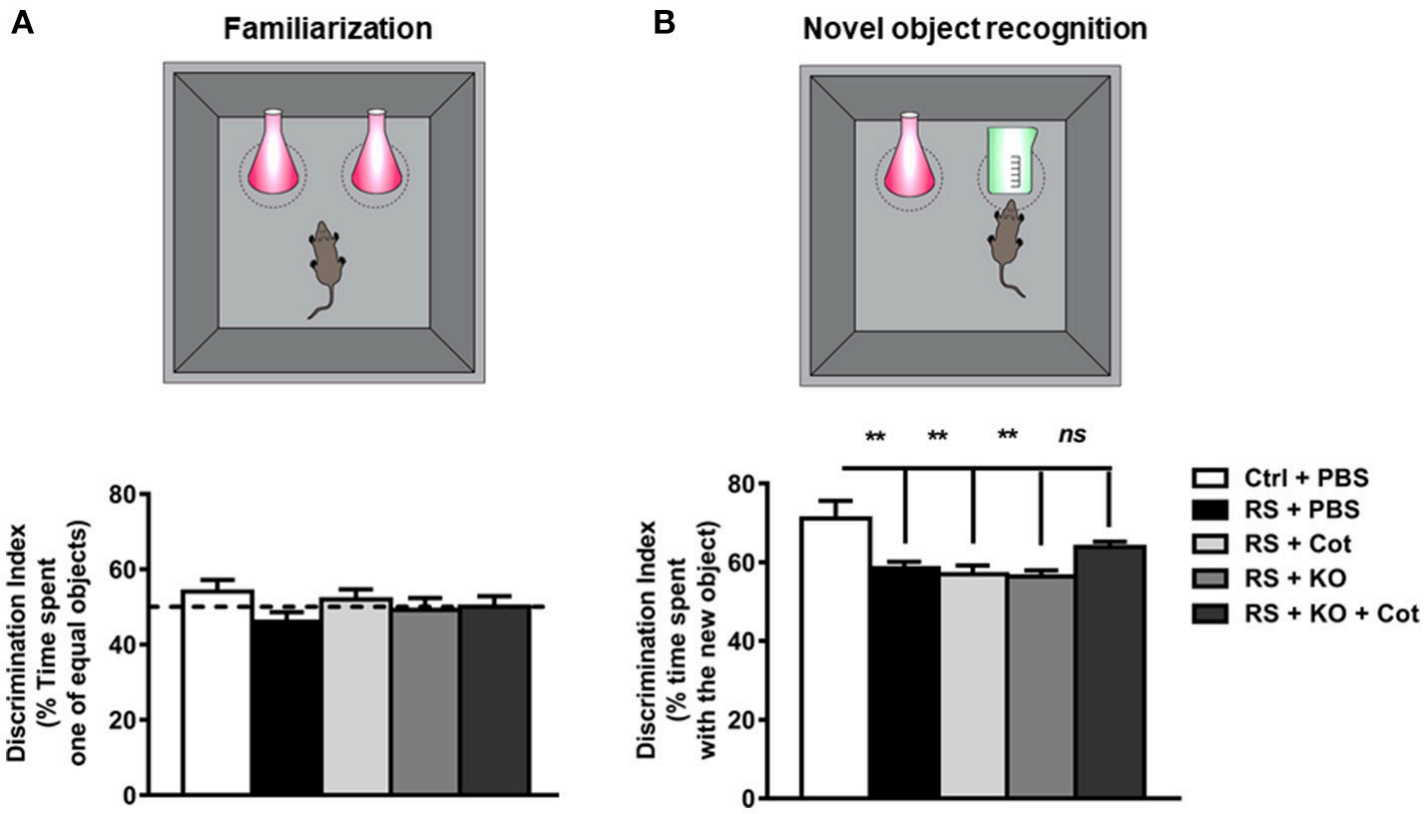
Co-treatment with cotinine decreased the restraint stress-induced deficit in recognition memory. After restraint and co-treatment with vehicle (PBS), cotinine (Cot, 5 mg/kg) krill oil (KO, 143 mg/kg) or Cot plus KO, mice were tested for locomotor activity in the open field test and next day mice were tested for recognition memory in the novel object recognition test (NOR). **(A)** Familiarization: mice were individually exposed to two identical objects. **(B)**, Novel object recognition step: after 30 min of rest, mice were exposed to one of the old objects and a new object. Chronic restraint stress impaired novel object recognition. Co-treatment with KO plus Cot preserved recognition memory abilities in the stressed mice to levels non-significantly different from control non-stressed mice. **P < 0.01.

To assess whether co-treatment with cotinine during RS influenced recognition memory, mice were tested in the novel object recognition NOR test. Non-significant differences were found between non-stressed and restrained mice in the familiarization step of the task, and all mice explored each of the two objects almost 50% of the time, no showing a preference for any of them (Figure 5A). However, when mice were exposed to one old object and a new object in the arena, one-way ANOVA analysis revealed significant differences between groups on recognition memory [*F*_(4, 48)_ = 4.29, *P* = 0.0049]. Multiple comparison tests showed a significant difference in the time spent with the new object between the vehicle-treated non-restrained mice and the vehicle-treated restrained mice (*P* < 0.05). However, restrained mice treated with cotinine plus KO showed no differences in recognition working memory reflected as a similar level of preference for the new object than vehicle-treated non-restrained mice (*P* > 0.05) (Figure 5B).

### Analysis of Changes in Morphology and Cell Viability of GFAP+ Astrocytes

#### Cell Counting

To assess changes induced by RS and treatments on astrocyte architecture and numbers. The number of GFAP+ cells, GFAP IR and area fraction were investigated in the DG, one of the region more affected in those parameters by RS (Perez-Urrutia et al., 2017).

**Figure 6:**
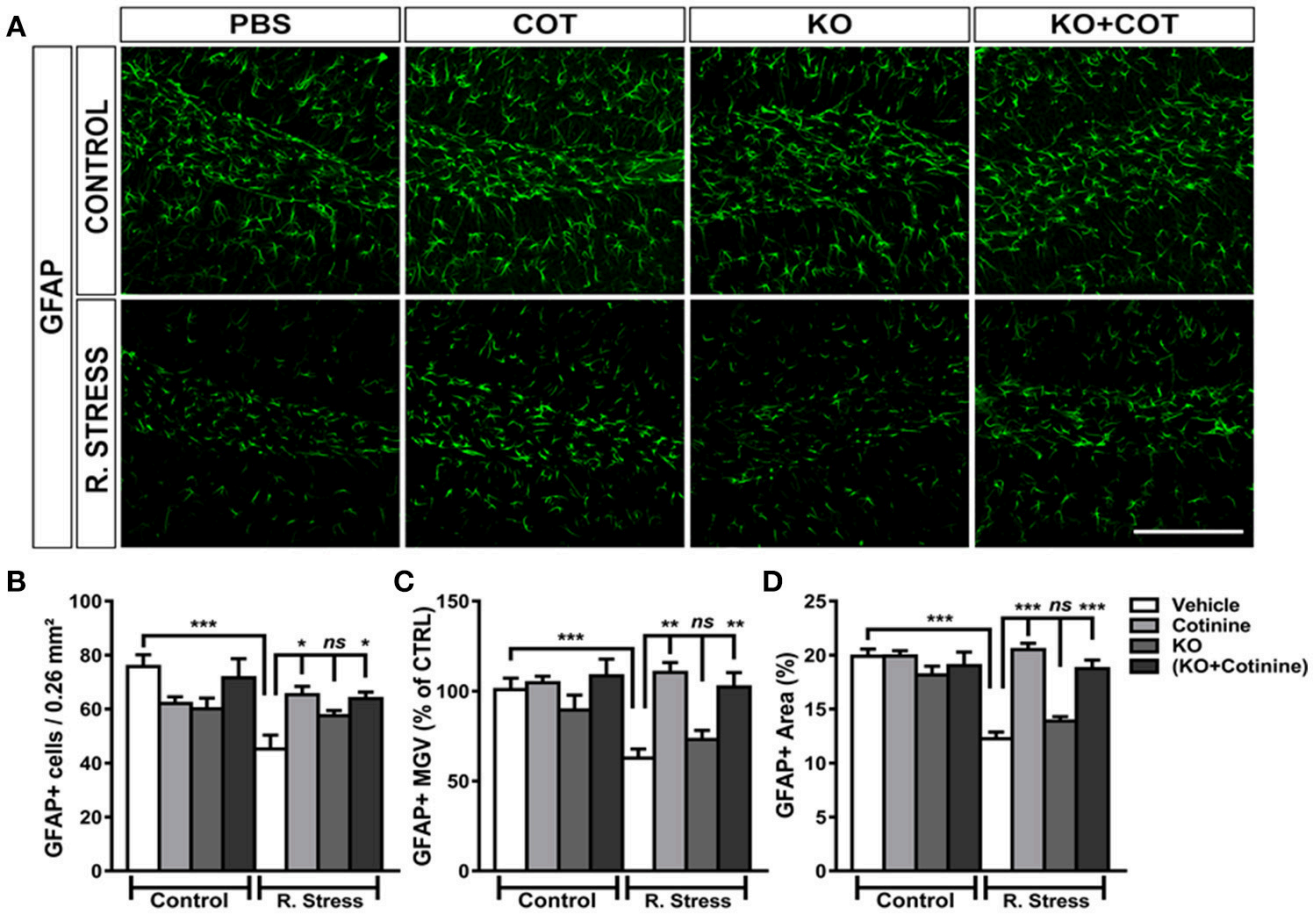
Analysis of the effect of cotinine plus krill oil on astrocytes in the dentate gyrus of the hippocampus. A figure representing the changes in cell GFAP+ cells numbers and morphology in the dentate gyrus region of the hippocampus in male mice subjected or not to restraint stress (R. Stress) **(A)**. Graph depicting the changes in the number of GFAP**+** IR cells **(B)**; main gray values (MGV) **(C)**; area of GFAP IR in the DG of vehicle-treated non-retrained mice or restrained (RS) mice, cotinine (Cot, 5 mg/kg) or Cot plus KO (143 mg/kg) (Cot + KO) **(D)**. *P < 0.05, **P < 0.01, ***P < 0.001.

Multiple comparison tests revealed no significant effects of treatments in GFAP IR in the non-stressed mice, but significant differences in the restrained mice. One-way ANOVA analysis of GFAP+ cells in the DG showed a significant effect of treatments on the number of GFAP+ cells in the DG [*F*_(7, 46)_ = 4.88, *P* = 0.0004]. GFAP+ cell densities in the DG were significantly reduced in the vehicle-treated restrained mice when compared to vehicle-treated non-stressed mice (*P* < 0.001). Treatment with KO alone did not induced changes in the number of GFAP+ cell in the KO-treated restrained mice, when compared to vehicle-treated restrained mice. However, a substantial increase of GFAP+ IR cells was observed in the cotinine-treated restrained mice or KO plus cotinine, when compared to vehicle-treated restrained mice (*P* < 0.05) (Figures 6A,B).

#### Mean Gray Value

One-way ANOVA analysis of mean GFAP IR in the GFAP+ cells in the DG showed a significant effect of treatments on GFAP IR intensity in the DG [*F*_(7, 33)_ = 5.10, *P* = 0.0005]. Multiple comparison tests revealed no significant effects of treatments on the mean GFAP IR in the non-stressed mice. However, a significant decrease in the mean IR intensity was found in the vehicle-treated restrained mice group when compared to vehicle-treated non-restrained mice (*P* < 0,05). Nonsignificant changes in GFAP IR were found in the KO-treated restrained mice when compared to the vehicle-treated restrained mice (*P* > 0.05). On the other hand, a significant increase of IR intensity was found in the cotinine-treated restrained mice when were compared to vehicle-treated restrained mice (*P* < 0.01). Also, there was a significant increase in GFAP IR in the KO plus cotinine-treated restrained mice when compared to vehicle-treated restrained mice (*P* < 0.01; Figure 6C).

#### Area Fraction

The analysis of the percent area fraction occupied by GFAP+ cells revealed significant effects of treatments in the DG [*F*_(7, 34)_ = 17.28, *P* < 0.0001]. Multiple comparison analysis showed that vehicle-treated restrained mice show a significant decrease of the GFAP+ IR area in comparison to non-stressed vehicle-treated mice (*P* < 0,001). Nevertheless, a significant increase in the GFAP+ IR fraction area was found in the cotinine and KO plus cotinine-treated restrained mice when compared to vehicle-treated restrained mice (*P* < 0.001) in the DG (Figure 6D).

## Discussion

Chronic immobilization or reduced mobility stress can result from obesity, paralysis induced by vascular events such as stroke, spinal cord injury, advanced age, and many neurodegenerative conditions such as arthrosis, and ataxia. These events result in depression and cognitive impairment in the affected individuals. RS is a broadly used model of stress-induced depressive-like behavior (Buynitsky and Mostofsky, 2009). Prolonged RS results in morphological changes in the brain such as retraction of processes in hippocampal neurons and astrocytes (Magarinos et al., 1997; McEwen et al., 1997), neuroinflammation (Bauer et al., 2001; de Andrade et al., 2012; Tymen et al., 2013), and cognitive deficits (Thorsell et al., 2000; Abidin et al., 2004; Conrad et al., 2004; Cherian et al., 2009; Mika et al., 2012) and depressive-like behavior in rodents (Buynitsky and Mostofsky, 2009; Chiba et al., 2012). It has been shown that cotinine administered before and after RS, reduces the depressive-like behavior, synaptic deficits, astrocyte alterations, and cognitive impairment in mice (Grizzell et al., 2014; Grizzell and Echeverria, 2015; Perez-Urrutia et al., 2017). In this study, it was investigated the effect of co-treatment with cotinine combined with KO, during chronic RS, on the development of depressive-like behavior and cognitive impairment induced by chronic stress in mice. RS provoked a decrease in recognition memory abilities and depressive-like behavior in the mice, however, cotinine plus KO prevented these behavioral changes. These results showed a synergistic beneficial effect of both cotinine and KO in preserving mood stability and cognitive abilities under conditions of chronic RS.

At the neurochemical level, chronic stress induces a deficit in glutamatergic neurotransmission by mechanisms involving a decrease of NMDA (N-Methyl-D-aspartate) and AMPA (α-amino-3-hydroxy-5-methyl-4-isoxazolepropionic acid) receptors in the postsynaptic site in the prefrontal cortex and the hippocampus, two brain regions that are fundamental for mediating declarative and working memory abilities. This reduction in the number of synaptic glutamate receptors induces a decrease in the activity of brain networks controlling stress behavior including the prefrontal cortex-amygdala-hippocampus pathways. Some evidence suggests that loss of glutamate receptors in neurons of the prefrontal cortex after repeated stress in rats, it is due to increased ubiquitin–proteasome-dependent degradation of these receptors (Joels et al., 2004; Yuen et al., 2012). Furthermore, previous studies using rodent models of chronic stress found a reduced proliferation of glial progenitor cells, and a decrease of GFAP+ cells in several brain regions, including the hippocampus and prefrontal cortex in rats. In rats, glucocorticoids can diminish the expression of GFAP in the PFC, resulting in >20% reduction in GFAP expression that was accompanied by a decrease of the GFAP mRNA levels (Zschocke et al., 2005). Also, chronic RS inhibits the glutamate uptake by astrocytes enhancing excitotoxicity and long-term depression (Yang et al., 2005). Furthermore, some evidence shows that rats exposed to early-life stress have a decrease in astrocytes levels in the frontal cortex in adulthood, indicating a long-term effect of stress on glial cells development (Leventopoulos et al., 2007). It is reasonable to propose that a deficit in astrocyte's function plays a crucial role in the higher susceptibility to PTSD observed in persons with a previous history of child abuse.

We have previously found a protective effect of intranasal cotinine administered against RS-induced astrocytes decrease. In this study, we found that co-treatment of mice with oral cotinine plus KO prevented the decrease in the number and complexity of astrocytes in the DG of mice subjected to RS. In this study, we observed a beneficial effect of cotinine and cotinine plus KO but not KO alone in preserving the number and arbor complexity of astrocytes under conditions of RS.

We have previously shown that, in the absence of stress, long-term cotinine treatment for up to 8 months did not induce significant differences in sensory-motor abilities or anxiety in mice (Zeitlin et al., 2012). Alike these results, no significant changes in locomotor activity in the mice treated with cotinine, KO or cotinine plus KO and subjected to RS were found. Thus, the superior effect of the combination of cotinine plus KO increasing the escape-oriented behavior in the FS test, cannot be explained by an increase in locomotor activity induced by the mix. The open field test is a good test to assess locomotor activity. However, the interpretation of the time spent in the center zone as a measure of anxiety has proven to be misleading and sometimes contradictory (Belzung, 2001a,b; Prut and Belzung, 2003). For example, it has been shown that many clinically effective non-benzodiazepine anxiolytics, except 5-HT1A agonists, these anxiolytic drugs exhibit extremely variable effects in the open field tests. Alike other anxiolytic drugs cotinine, KO or cotinine plus KO did not diminish the increased locomotor activity or time spent in the center zone during the first 10 min of the test, that is considered a measure of anxiety behavior after prolonged restraint stress.

It is appealing that comparable results were obtained in the behavioral parameters tested, with a more significant effect of the mix cotinine plus KO than the individual components in the mix. The connection between changes in astrocytes and depressive-like behavior has been reported before. For example, a previous study reported that the decrease in astrocytes numbers in the frontal cortex induced by L-alpha-aminoadipic acid provoked depressive-like behavior in rodents (Lee et al., 2013). This evidence demonstrated that astroglia ablation in the PFC is sufficient to prompt depressive-like behaviors comparable to the one induced by chronic RS. This data strongly suggests that the loss of astroglia may be a key factor contributing to the development of long-lasting depression (Lee et al., 2013).

The effect of cotinine in the mix preventing the effect of stress on mood can be the result of the action of cotinine as an anti-inflammatory compound inhibiting microgliosis and neuroinflammation as well as promoting neuronal and astrocyte survival throughout the activation of pro-survival cell signaling pathways.

**Figure 7:**
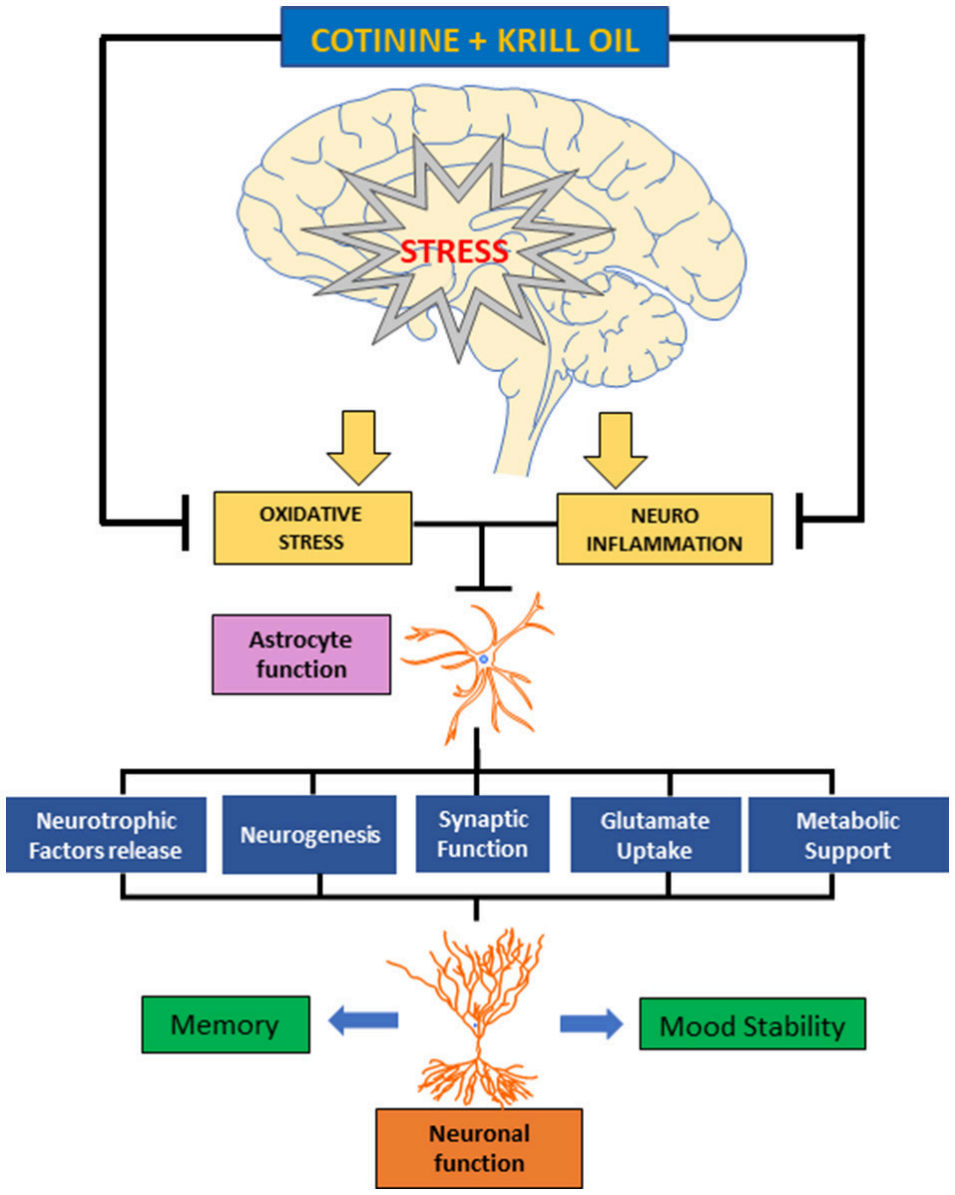
Diagram representing the effect of cotinine and krill oil preventing the effects of chronic stress on astrocyte and neuronal function and behavior. Cotinine plus KO may counteract the neuroinflammatory and oxidative processes triggered by chronic stress in the brain. This effect may prevent the reduction in astrocyte affecting their role supporting the neuronal plasticity processes that are required for working memory and mood stability affected by chronic stress.

Increased levels of astrocytes may provide neurons with more energy substrates, glutamate precursors, and neurotrophic factors. Also, astrocytes can decrease the toxic effect of the abnormal increase in glutamate release induced by corticosteroids at the presynaptic level, by uptaking the glutamate from the synaptic space. On the other hand, KO components such as omega-3 and astaxanthin can prevent oxidative stress and diminish the deleterious effects of stress and Neuroinflammation on brain function (Barros et al., 2014; Polotow et al., 2015; Figure 7).

A more detailed study of the effect on cotinine and KO on astrocyte function is guaranteed in the light of the present results.

## Conclusions

In this work, the effect of an oral formulation of cotinine plus KO to prevent the cognitive and depressive-like behavior induced by chronic restraint stress. The results show that at the dose tested, the cotinine plus KO prevented depressive-like behavior, memory impairment and the astrocytes decrease induced by RS, and suggests that this formulation may be useful in humans and non-primates mammals suffering from restraint stress due to aging or other pathological and traumatic conditions. Clinical studies are needed to confirm this hypothesis.

## Author Contributions

All authors listed have made a substantial, direct and intellectual contribution to the work, and approved it for publication.

### Conflict of Interest Statement

VE is the inventor of the patent (US 20100104504), for the use of cotinine for a post-traumatic stress disorder (University of South Florida, Veterans Affairs) and the provisional patent (US62459736) for the combination of cotinine plus antioxidants for treatment-resistant depression and correction of astrocytes functional deficit induced by depression and other neuropathological conditions (Veterans Affairs and the Universidad San Sebastian). The remaining authors declare that the research was conducted in the absence of any commercial or financial relationships that could be construed as a potential conflict of interest.

## Abbreviations

AMPA: α -amino-3-hydroxy-5-methyl-4-isoxazolepropionic acid
ANOVA: Analysis of variance
CRH: corticotrophin releasing hormone
FS: Forced swimming
GFAP: Glial fibrillary acidic protein
HPA: Hypothalamus-pituitary-adrenal
IR: Immunoreactivity
KO: Krill oil
MD: Major depression
MAOIs: Monoamine oxidase inhibitors
MGV: Mean gray values
NOR: Novel object recognition
OF: Open field
PBS: Phosphate buffered saline
PFC: Prefrontal cortex
PTSD: Posttraumatic stress disorder
ROI: Region of interest
SSRIs: Region serotonin-reuptake inhibitors
SNRIs: Serotonin-norepinephrine reuptake inhibitors
TBS: Tris-buffered saline
TBST: TBS with 0.1% Tween 20.
